# New‐Onset Myasthenia Gravis Mimics Spinal Cord Injury After Zenker's Diverticulum Repair

**DOI:** 10.1002/oto2.56

**Published:** 2023-06-08

**Authors:** Steffen E. Meiler, Tylin J. Siwemuke, Gregory N. Postma

**Affiliations:** ^1^ Department of Anesthesiology and Perioperative Medicine, Medical College of Georgia Augusta University Augusta Georgia USA; ^2^ Medical College of Georgia Augusta University Augusta Georgia USA; ^3^ Department of Otolaryngology, Medical College of Georgia Augusta University Augusta Georgia USA

**Keywords:** central cord syndrome, drug interactions with myasthenia gravis, icepack test, myasthenia gravis, postoperative myasthenia gravis complications

Myasthenia gravis (MG) is the most common autoimmune disease affecting the neuromuscular junction (NMJ). Untreated this disease can lead to profound skeletal muscle weakness and respiratory failure.^1^ Although rare, intraoperative events can precipitate the first clinical presentation of MG, potentially creating diagnostic uncertainty in the postoperative period, and requiring early recognition and management of this condition to prevent the possibility of life‐threatening complications in the immediate postoperative period.^2^ This project received Institutional Review Board approval, Medical College of Georgia, Augusta University # 611858.

## Discussion

A 60‐year‐old white male underwent endoscopic repair of a Zenker's diverticulum under general anesthesia. Medication administration included ampicillin and sulbactam prior to incision, lidocaine, propofol, and remifentanil for induction and maintenance of anesthesia (total intravenous anesthesia), rocuronium, ketoroloc, ondansetron, dexamethasone, and neostigmine and glycopyrrolate for reversal of neuromuscular blockade (NMB). Emergence from anesthesia, return of spontaneous ventilation and extubation was uneventful; however, during recovery, the patient began to complain of new‐onset upper and lower extremity weakness. Muscle strength grading using the Medical Research Council Manual Muscle Testing scale revealed upper extremity greater than lower extremity weakness (4− out of 5 vs 4 out of 5). The patient was only able to ambulate with 2‐person assistance and did not exhibit sensory deficits or difficulty urinating. The clinical features were suggestive of acute cervical spinal cord injury, or central cord syndrome, raising the concern that this devastating complication had resulted from prolonged neck extension during surgery. Central cord syndrome has previously been recognized as a complication of neck extension during surgical procedures or emergency intubations.^3^ Fortunately, emergency magnetic resonance imaging and computed tomography imaging were able to rule out spinal cord injury or other traumatic lesions. A focused repeat neurological examination uncovered mild bilateral ptosis, some weakness in eye closure, no diplopia, no dysarthria, and a normal cough. A bedside icepack test was applied to the eyelids for 2 minutes, which was strongly positive for MG and resulted in complete resolution of the ptosis. After additional clinical and laboratory testing, the postoperative diagnosis of new‐onset MG was confirmed, and the patient received appropriate treatment at our hospital.

In MG autoantibodies damage the nicotinic acetylcholine receptor (AChR) at the postsynaptic membrane and impair neuromuscular transmission. In this case, it is reasonable to assume that exposure to several intraoperative drugs further reduced the efficiency of neuromuscular transmission and unmasked this patient's previously undiagnosed disease. Specifically, the NMJ in MG is exquisitely sensitive to nondepolarizing neuromuscular‐blocking agents (eg, rocuronium), different classes of antibiotics (eg, aminoglycosides, ampicillin), possibly local anesthetics (eg, lidocaine), and glucocorticoids_._ ^1^ Our patient received the above medications during surgery, which are postulated to have various pre‐ and postsynaptic effects, negatively impacting the availability of acetylcholine and AChR function. In addition, more recently published work has demonstrated that reversal of NMB with neostigmine and glycopyrrolate tends to be incomplete with a reported incidence of residual NMB of up to 63.5% at tracheal extubation and 56.5% at arrival in the postanesthesia care unit.^4^ Any degree of residual NMB would have contributed to this patient's clinical picture. These considerations highlight that in a surgical patient with known MG, the perioperative pharmacology is complex, requires careful preoperative planning, and aims to preserve the function of the compromised NMJ. Of note, the ice pack test used in this patient increases the availability of acetylcholine in the NMJ through cold inhibition of the acetylcholine‐degrading enzyme acetylcholinesterase, which explains the high diagnostic performance of this bedside test for MG.^5^

This report presents a rare case of new‐onset MG diagnosed in the immediate postoperative period after a Zenker's repair. It demonstrates that intraoperative events can unmask MG during the subclinical stage of the disease, lead physicians to other diagnostic considerations because of the general unfamiliarity with this complication in the postoperative period, and challenges surgeons and anesthesiologists to be aware of this potentially life‐threatening clinical scenario.

## Author Contributions

**Steffen E. Meiler**, directed the anesthesia care, diagnosed the patient postoperatively, and drafted the manuscript; **Tylin J. Siwemuke**, performed the literature search and assisted in writing the manuscript; **Gregory N. Postma**, directed the surgical care and contributed to the final manuscript.

## Disclosures

### Competing interests

No conflicts of interest to report.

### Funding source

None.
